# Sociodemographic and clinical factors of women with HPV and their association with HIV^1^

**DOI:** 10.1590/0104-1169.3364

**Published:** 2015

**Authors:** Joice Gaspar, Silvana Maria Quintana, Renata Karina Reis, Elucir Gir

**Affiliations:** 2 Master's student, Escola de Enfermagem de Ribeirão Preto, Universidade de São Paulo, WHO Collaborating Centre for Nursing Research Development, Ribeirão Preto, SP, Brazil. Scholarship holder, Coordenação de Aperfeiçoamento de Pessoal de Nível Superior (CAPES), Brazil; 3 PhD, Associate Professor, Faculdade de Medicina de Ribeirão Preto, Universidade de São Paulo, Ribeirão Preto, SP, Brazil; 4 PhD, Professor, Escola de Enfermagem de Ribeirão Preto, Universidade de São Paulo, WHO Collaborating Centre for Nursing Research Development, Ribeirão Preto, SP, Brazil; 5 PhD, Full Professor, Escola de Enfermagem de Ribeirão Preto, Universidade de São Paulo, WHO Collaborating Centre for Nursing Research Development, Ribeirão Preto, SP, Brazil

**Keywords:** Papillomavirus Infections, Women, HIV

## Abstract

**OBJECTIVE:**

to identify the association between HIV-seropositive or HIV-seronegative status and the sociodemographic and clinical variables of women with genital HPV infection.

**METHOD:**

cross-sectional, retrospective study in a reference service in Ribeirão Preto. A total of 824 women undergoing HIV testing who had high or low grade cervical intraepithelial lesions or condylomatous genital lesions caused by HPV were studied. The chi-square test and logistic regression analysis with the calculation of the odds ratio and a confidence interval of 95% were conducted to verify the association.

**RESULTS:**

a higher probability of seropositivity was identified for non-white women; with low education; widowed; who consumed alcohol, tobacco or illicit drugs; with hepatitis C; who had multiple partners; and that worked as prostitutes.

**CONCLUSION:**

the increasing impairment of women due to sexually transmitted infections, considering the influence of the socioeconomic and behavioral context on the course of these infections, highlights the importance of public policies that establish intervention strategies involving the prevention, early diagnosis and timely treatment of these diseases, so that there is the promotion of quality of life in this population.

## Introduction

Infection with the human papillomavirus (HPV) is a public health problem, being considered the most common sexually transmitted infection (STI). It is estimated that approximately 600 million people have HPV worldwide and that about 75-80% of the population will acquire the virus at some point in life^(^ 1 ^)^.

Brazil is a world leader in incidences of HPV, with women between 15 and 25 years of age being the population most affected. Although this infectious disease also extends to males, it is believed that the number of registered cases is smaller due to the low demand of men for urology services, a factor related to prejudice and a lack of information^(^ 2 ^)^.

HPV infection has been associated with the human immunodeficiency virus (HIV), suggesting a greater chance of developing low (LSIL) and high grade (HSIL) cervical intraepithelial lesions in women living with HIV, due to their immunosuppression^(^ 3 ^-^ 5 ^)^. It is noteworthy that the prevalence of these lesions in HIV seropositive women with a CD4+ counts below 200 cells per µl and a viral load greater than 10,000 copies per mL is three times higher when compared to seronegative women^(^ 6 ^-^ 7 ^)^.

In addition to the greater chance of developing cervical intraepithelial lesions, women living with HIV present significantly longer persistence of HPV infections than those without the virus. Some authors have associated this predisposition with the fact that these women suffer lower levels of CD4+ T lymphocytes and increased viral load levels^(^ 8 ^)^, in addition to presenting a greater number of cervical samples of HPV viral DNA^(9-10) ^and having a higher incidence of high oncogenic risk virus types^(^ 11 ^-^ 13 ^)^.

Furthermore, HIV infection alters the natural history of HPV infection, with lower rates of regression from LSIL and higher risk of progression to HSIL and invasive lesions resistant to treatment, making more interventions and monitoring necessary^(^ 3 ^,^ 5 ^,^ 10 ^)^. A study on the association between HPV infection and women living with HIV found that this issue is particularly relevant in relation to the establishment of appropriate prevention strategies and for the treatment of patients, requiring prior knowledge of the epidemiology and pathogenesis of HPV infection in the population of HIV seropositive women^(^ 5 ^)^.

Therefore, due to the relevance of HPV/HIV co-infection, the Centers for Disease Control and Prevention (CDC) has considered the precursor lesions of cervical cancer in the classification of HIV infection since 1993. Women with HIV infection who present LSIL or HSIL are classified as symptomatic (category B of the infection). Those that present cervical cancer are classified as AIDS carriers (category C)^(^ 14 ^)^.

Considering the above information, the aim of this study was to analyze the association between HIV-seropositive or HIV-seronegative status and the sociodemographic and clinical variables of women with genital HPV infection. The intention was to contribute to the production of theoretical knowledge capable of supporting the design and implementation of public policies that enhance the management of harm resulting from both infections, through prevention and treatment strategies and the organization of the services and health practices.

## Methods

This was a cross-sectional, retrospective study, conducted in a specialized service for Infectious Diseases in Gynecology and Obstetrics at a Hospital located in a large municipality of the state of São Paulo.

The study population consisted of 824 women who were registered and treated in the specialized service in question, who met the following inclusion criteria: to present genital HPV infection with diagnosis of LSIL, HSIL or condylomatosis (vulvar, vaginal, cervical and perianal); to have complete records in the electronic database of the study sector with information concerning the anamnesis performed in the first consultation and information relating to the consultations and returns attended, and to have performed the serological test for HIV.

For the data collect, a structured form was developed specifically for this study, with the form and content having been validated by three specialists in HPV infection. The variables included were: 1. Sociodemographic: age, race, marital status, education, economic status, alcohol use, tobacco use, illicit drug use, and sexual behavior (age at first intercourse, total number of sexual partners, number of sexual partners in the previous year, prostitution, and sexual orientation) and 2. Clinical: serological tests for HIV, hepatitis B and C, and syphilis, and time since HIV diagnosis.

**Figure 1 -:**
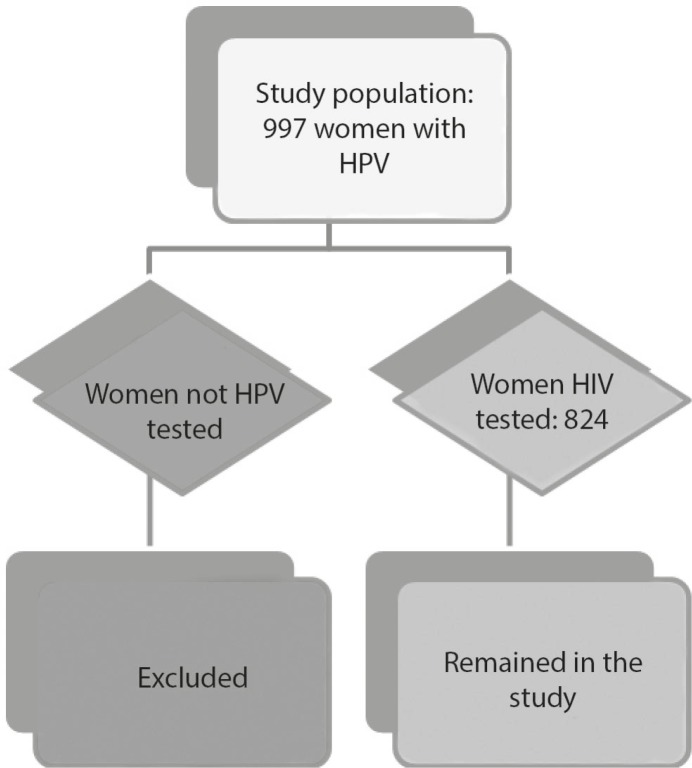
Flowchart presenting the definition of the study population

For the sociodemographic and clinical characterization a search of the electronic database of the study site was performed between March and October 2012. Through this search women were selected who had, as a reason for attending the consultation, at least one of the following items in their records: vulvar condyloma, vaginal condyloma, cervical condyloma, perianal condyloma, and cervical intraepithelial neoplasia (CIN) grade I, II or III. A total of 977 records were found, corresponding to the period from 09/10/1986 to 23/10/2012. Of this total, 153 records had no serological test for HIV and were excluded from the analysis, resulting in the inclusion of 824 women (Figure 1).

The database was organized in spreadsheets using the Microsoft Excel 2010 program, with data validation after double entry. The study population was characterized by descriptive statistics and the data were processed and analyzed using the Statistical Package for the Social Sciences (SPSS) version 16.0 for Windows.

To verify the association between HIV-seropositive or HIV-seronegative status and the sociodemographic and clinical variables in women with genital HPV infection, the data were submitted to the chi-squared test. The quantification of these associations was measured using simple logistic regression models, calculating the crude Odds ratio (OR) with respective confidence intervals of 95%. Statistical analyzes were performed using the SAS^(r)^ 9.0 statistical software. A p-value of less than 0.05 was considered significant.

For the logistic regression, "HIV-seropositive" and "HIV-seronegative" were denominated as the dichotomous dependent variables and all sociodemographic and clinical variables as independent variables. The sociodemographic variables considered in this study were: age (classified into seven age groups), ethnicity (dichotomized as white or non-white), marital status (single, stable union, separated, and widowed) education (classified in four groups of years of education), economic situation (with income, without income, and other), alcohol use (dichotomized as yes or no), smoking (dichotomized as yes or no), use of illicit drugs (dichotomized as yes or no) and sexual behavior, which included age of first sexual intercourse (classified into three age groups), total number of sexual partners (01|-|05, 05|-|10 and >10), number of sexual partners in the last year (<3 and >3), prostitution (dichotomized as yes and no) and sexual orientation (dichotomized as heterosexual and not heterosexual). Regarding the clinical variables, serology for hepatitis B and C and syphilis were prominent, all dichotomized into positive and negative.

For the application of the statistical model, the ethnicity variable, which was categorized into four groups, was dichotomized as white and non-white, due to the low numbers found in some of the options of the table, so as to make the analysis feasible.

The answers "no information" for each clinical and socio-demographic variable were considered missing and not entered in the analysis. In the serology for hepatitis B, hepatitis C, and syphilis items the answer "not performed" as well as answer "no information" were considered missing. In the total number of sexual partners item only one woman answered "never had sexual relations", therefore, it was excluded from the analysis, yielding an *n* of 823 women for this item.

The study was reviewed and approved by the Research Ethics Committee of the Ribeirão Preto College of Nursing at the University of São Paulo under process No. 1303/2011, and followed the recommendations of Resolution 196/96 of the National Health Council. Confidentiality and anonymity of information were assured for all participating women. For the consultation of the electronic database a waiver of the need to obtain informed consent was requested, considering that only secondary data obtained from the study of already collected and recorded information was used.

## Results

Of the 824 women, 326 (39.56%) were between 20 and 29 years of age, 531 (64.68%) were white, 361 (43.81%) had completed five to eight years of education, 486 (58 98%) were in stable unions, 430 (52.18%) had no fixed income, and 711 (86.28%) did not consume alcohol, 576 (69.90%) tobacco and 713 (86.52%) illicit drugs. Of the total, 152 were HIV-seropositive (18.45%) and 672 HIV-seronegative (81.55%), 334 (40.53%) had HSIL, 289 (35.07%) condyloma and 201 (24.39%) LSIL with lesions caused by HPV.

When evaluating, by means of logistic regression, the association between HIV-seropositive or HIV-seronegative status and the sociodemographic variables, there was a higher probability of seropositivity for non-white women (p<0.01; OR=1.990; CI=1.392-2.843); who studied 0-4 years (p<0.01; OR=4.384; CI=1.706-11.266) or 5-8 years (p<0.01; OR=2.530; CI=1.051-6.093); were widowed (p<0.01; OR=4.223; CI=1.869-9.542); consumed alcohol (p=0.0013; OR=2.120; CI=1.333-3.374), smoked (p<0.01; OR=2.389; CI=1.660-3.437), or used illicit drugs (p<0.01; OR=2.936; CI=1.882-4.580) (Table 1).

**Table 1 -:** Association and logistic regression between the sociodemographic variables and HIV status of women with genital HPV infection (n=824) treated at a university hospital in São Paulo state, Ribeirão Preto-SP, Brazil, 1986-2012

		HIV		Missing	p-value*	OR^†^	CI^‡^ (95%)
		Negative n (%)	Positive n (%)				
Age group (years)				0	<0.01		
	<16	8 (0.97)	2 (0.24)			1.000	Reference
	16|–|19	30 (3.64)	9 (1.09)			1.200	(0.215 ; 6.696)
	20|–|29	288 (34.95)	38 (4.61)			0.528	(0.108 ; 2.578)
	30|–|39	206 (25.00)	43 (5.22)			0.835	(0.171 ; 4.070)
	40|–|49	89 (10.80)	39 (4.73)			1.753	(0.356 ; 8.635)
	50|–|59	38 (4.61)	12 (1.46)			1.263	(0.235 ; 6.777)
	≥ 60	13 (1.58)	9 (1.09)			2.769	(0.473 ; 16.213)
Ethnicity (cited)				3	<0.01		
	White	453 (55.18)	78 (9.50)			1.000	Reference
	Non-white	216 (26.31)	74 (9.01)			1.990	(1.392 ; 2.843)
Education (years)				13	<0.01		
	0|–|4	66 (8.01)	31 (3.76)			4.384	(1.706 ; 11.266)
	5|–|8	284 (34.47)	77 (9.34)			2.530	(1.051 ; 6.093)
	9|–|11	253 (30.70)	38 (4.61)			1.402	(0.565 ; 3.477)
	≥ 12	56 (6.80)	6 (0.73)			1.000	Reference
Marital status				9	<0.01		
	Single	200 (24.27)	51 (6.19)			1.000	Reference
	Stable Union	413 (50.12)	73 (8.86)			0.693	(0.467 ; 1.029)
	Separated	38 (4.61)	13 (1.58)			1.342	(0.666 ; 2.704)
	Widowed	13 (1.58)	14 (1.70)			4.223	(1.869 ; 9.542)
Economic situation				18	0.5182		
	With income	127 (15.41)	33 (4.00)			1.000	Reference
	Without income	348 (42.23)	82 (9.95)			0.907	(0.577 ; 1.425)
	Other	181 (21.97)	35 (4.25)			0.744	(0.439 ; 1.260)
Alcohol use				10	0.0013		
	Yes	72 (8.74)	31 (3.76)			2.120	(1.333 ; 3.374)
	No	591 (71.72)	120 (14.56)			1.000	Reference
Tobacco use				5	<0.01		
	Yes	174 (21.12)	69 (8.37)			2.389	(1.660 ; 3.437)
	No	494 (59.95)	82 (9.95)			1.000	Reference
Drug use				5	<0.01		
	Yes	68 (8.25)	38 (4.61)			2.936	(1.882 ; 4.580)
	No	599 (72.69)	114 (13.83)			1.000	Reference
Type of lesion					<0.01		
	Low-grade cervical intraepithelial lesion	154 (18.69)	47 (5.70)			0.953	(0.632 ; 1.438)
	Condyloma	265 (32.16)	24 (2.91)			0.283	(0.174 ; 0.460)
	High-grade cervical intraepithelial lesion	253 (30.70)	81 (9.83)			1.000	Reference

* p-value refers to the Chi-Squared test

† Crude Odds Ratio

‡ Confidence Interval

Of the 824 women, eight (0.97%) were seropositive for hepatitis B, 21 (2.55%) for hepatitis C and six (0.73%) for syphilis, 437 (53.03%) had their first sexual relations before age 16, 619 (75.86%) had one to five sexual partners over the lifetime, 777 (95.69%) had less than three sexual partners in the previous year, 793 (96.24%) had never resorted to prostitution, and 809 (99.14%) were heterosexual.

Regarding the association between HIV seropositivity or seronegativity and the clinical and sexual behavior variables, there was a higher probability of seropositivity for women with hepatitis C (p<0.01; OR=10.529; CI=4.160-26.647); that had 5 to 10 (p<0.01; OR=1.985; CI=1.207-3.264) or more than 10 (p<0.01; OR=3.487; CI=2.170-5.602) sexual partners over the lifetime; and those that had been prostitutes (p=0.0039; OR=3.699; CI=1.434-9.540) (Table 2).

**Table 2 -:** Association and logistic regression between the HIV status and the clinical and sexual behavior variables of women with genital HPV infection (n=824) treated at a university hospital in São Paulo state, Ribeirão Preto-SP, Brazil, 1986-2012

		HIV			Missing		p-value^‡^	OR^§^	CI^||^ (95%)
		Negative n (%)	Positive n (%)		N/P*	N/I^†^			
Hepatitis B serology					10	27	0.1148		
	Positive	5 (0.61)	3 (0.36)					3.023	(0.714 ; 12.809)
	Negative	650 (78.88)	129 (15.66)					1.000	Reference
Hepatitis C serology					25	45	<0.01		
	Positive	7 (0.85)	14 (1.70)					10.529	(4.160 ; 26.647)
	Negative	616 (74.76)	117 (14.20)					1.000	Reference
Syphilis serology					8	27	0.9900		
	Positive	5 (0.61)	1 (0.12)					1.000	Reference
	Negative	651 (79.00)	132 (16.02)					1.014	(0.117 ; 8.749)
First sexual relations (years)						0	0.9293		
	< 16	355 (43.08)	82 (9.95)					1.155	(0.543 ; 2.457)
	16|–|20	272 (33.01)	61 (7.40)					1.121	(0.520 ; 2.416)
	> 20	45 (5.46)	9 (1.09)					1.000	Reference
Total number of sexual partners (n=823)						7	<0.01		
	01|–|05	529 (64.83)	90 (11.03)					1.000	Reference
	05|–|10	77 (9.44)	26 (3.19)					1.985	(1.207 ; 3.264)
	>10	59 (7.23)	35 (4.29)					3.487	(2.170 ; 5.602)
Sexual partners in the previous year						12	0.4813		
	< 3	636 (78.33)	141 (17.36)					1.000	Reference
	>3	27 (3.33)	8 (0.99)					1.337	(0.595 ; 3.004)
Prostitution						13	0.0039		
	Yes	10 (1.21)	8 (0.97)					3.699	(1.434 ; 9.540)
	No	652 (79.13)	141 (17.11)					1.000	Reference
Sexual orientation						8	0.4910		
	Heterosexual	660 (80.88)	149 (18.26)					1.000	Reference
	Not heterosexual	5 (0.61)	2 (0.25)					1.772	(0.340 ; 9.221)

Not performed

† No Information

‡ p-value refers to the Chi-Squared test

§ Crude Odds Ratio

|| Confidence Interval

## Discussion

In this study there was a statistically significant association between ethnicity and HIV seropositivity, suggesting that non-white women have 1.990 times higher probability for HIV positivity (p<0.01; OR=1.990; CI=1.392-2.843) when compared to white women.

Regarding the risk condition of non-white women for HIV, a study conducted in the state of São Paulo, which analyzed characteristics related to vulnerability of HIV-seropositive women according to skin color, noted disadvantages of black women in exposure to health risks and restrictions of adequate resources for their care, highlighting lower socioeconomic status, lower levels of education, and worse housing conditions for this group^(^ 15 ^)^.

The relationship between ethnicity and HIV/AIDS is contained in the historical, cultural, political and ideological constructs^(16).^ The exposure of black people to the cumulative processes of social disadvantage influences their greater or lesser access to health services, material goods, education, housing, public goods, and information, leading to higher incidence of HIV infection in this population^(^ 16 ^-^ 17 ^)^.

A statistically significant association was identified between low education and HIV seropositivity (p<0.01), indicating a 4.384 times higher probability of being HIV-seropositive for women who have 0-4 years of education (OR=4.384; CI=1.706-11.266) and 2.530 times higher probability for women who have 5-8 years of education (OR=2.530; CI=1.051-6.093).

Education is a determining factor in social vulnerability. Knowledge mediates attitudes that will benefit or not the risk perception regarding the cervical cancer precursor lesions^(^ 18 ^)^. Access to healthcare services and adherence to the HIV/AIDS treatment are also mediated by education, which impacts on the comprehension of the therapy, due to difficulties in the interpretation of the information provided by the health team and in the recognition of the importance of performing the treatment correctly^(^ 19 ^)^.

Regarding marital status, although this study indicated a higher frequency of women in stable unions, affected by HPV, the test of association between HIV status and marital status (p<0.01; OR=4.223; CI=1.869-9.542) suggested a 4.223 times greater probability of seropositivity for widowed women.

Regarding the fact that being widowed was considered predisposing for HIV-seropositivity, a study performed in an outpatient clinic of a reference center in the STI area, located in São Paulo, which evaluated sexuality and reproductive health of women living with HIV/AIDS, claimed that this data was to be expected, since many of these women became widows because their partners had AIDS^(^ 20 ^)^.

In relation to the use of alcohol, illicit drugs and tobacco, women who consumed alcohol presented a 2.120 times higher probability of being HIV seropositive (p=0.0013; OR=2.120; CI=1.333-3.374), those who smoked, a 2.389 times greater probability (p<0.01; OR=2.389; CI=1.660-3.437), and those who used illicit drugs 2.936 times greater probability (p<0.01; OR=2.936; CI=1.882-4.580) when compared to those did not use these substances.

Drug users generally start their sexual life earlier when compared to non-users, use condoms less, pay for sex and have involvement in prostitution^(^ 21 ^)^. A study by the Center for Epidemiological Research into AIDS^(^ 22 ^)^ observed that the consumption of alcohol and drugs, with the exception of cocaine and crack cocaine, stimulates sexual activity, because soon after consumption the impression of the users is that relating to the other person becomes easier, the libido increases and the sexual performance improves.

Regarding co-infections, women with hepatitis C had a 10.529 times higher probability of being HIV-seropositive (p<0.01; OR=10.529; CI=4.160-26.647) than those who did not have the infection. It is known that their etiological agents share the same transmission mechanisms, i.e. the parenteral, sexual and vertical routes, which explains the high frequency of simultaneous infection by both viruses^(^ 23 ^)^.

In relation to the sexual behavior, women who had five to 10 partners throughout the lifetime had a 1.985 higher probability of having HIV (p<0.01; OR=1.985; CI=1.207-3.264) than those who had one to five partners, and those who had more than 10 partners had a 3.487 higher probability of having the infection (p<0.01; OR=3.487; CI=2.170-5.602) when compared to the same group.

The multiplicity of sexual partners is classified as major risk factor for the acquisition of STIs, given that women with such behavior have a greater possibility of contact with different viral types with each contact with a new sexual partner^(^ 24 ^)^.

Considering prostitution, a 3.699 times higher probability of having HIV was verified in the women who reported having offered sexual services (p=0.0039; OR=3.699; CI=1.434-9.540).

A study that characterized the population of active sex workers in a large municipality in the state of São Paulo, considered that they are more susceptible to sexually transmitted infections by pathogens, including HIV and HPV, than the general population, due to factors directly related to the prostitution, such as the greater number of partners and lack of condom use, and due to socioeconomic factors such as low levels of education and low purchasing power. Therefore, it is necessary that the healthcare services start to pay more attention to this population, both in terms of the provision of preventive programs, as well as the development of new investigations to that would provide better knowledge about the specific risk factors of this group for STIs^(^ 25 ^)^.

The targeting of actions and interventions of healthcare professionals should be based on the context of social and economic inequalities experienced by the population, especially by women, with possible reflections on the conditions of access and permeability to the healthcare services, both for prevention and for the timely diagnosis and ongoing therapy.

## Conclusion

A higher possibility of seropositivity was identified in this study for the following women with HPV: non-white; with low education levels; widowed; who consumed alcohol, tobacco or illicit drugs; with hepatitis C; who had multiple partners; and that had worked as prostitutes.

The social and economic inequalities experienced by these women reveal numerous consequences resulting from the pathology and the gender disparity, related to stigma and prejudice, with impact in their social, family, emotional and sexual relationships.

Considering the increasing involvement of women in the STIs and the strong influence of the socioeconomic context on the route of these infections, the importance of public policies that establish appropriate strategies for prevention, early diagnosis and treatment are highlighted, aiming for the promoting of quality of life in this population.
